# Prevalence, risk factors, and molecular epidemiology of simian foamy virus in free-living Macaca fascicularis at human–macaque interfaces in eastern Thailand

**DOI:** 10.14202/vetworld.2026.3995-4007

**Published:** 2026-09-11

**Authors:** Daraka Tongthainan, Phairot Phromwat, Phawaran Meetipkit, Supriya Kongpom, Poramaporn Kromsuriyasak, Wachirawich Nimitpan, Suchawan Pornsukarom

**Affiliations:** 1 Faculty of Veterinary Medicine, Rajamangala University of Technology Tawan-ok, Si Racha, Chonburi, Thailand.; 2 Department of National Parks, Wildlife and Plant Conservation, Bangkok, Thailand.

**Keywords:** Chonburi Province, human–macaque interface, long-tailed macaque, molecular epidemiology, One Health, risk factors, simian foamy virus, zoonotic transmission

## Abstract

**Background and Aim:**

Long-tailed macaques (*Macaca fascicularis*) are widely distributed in Thailand and often inhabit areas where they come into contact with humans, creating opportunities for zoonotic pathogen transmission. Simian foamy virus (SFV), a retrovirus of the subfamily Spumaretrovirinae, is of particular One Health relevance; however, site-specific epidemiological and molecular information from areas with frequent human–macaque interactions in Thailand remains limited. This study investigated the prevalence, associated risk factors, and molecular epidemiology of SFV infection in free-living long-tailed macaques at human–macaque interfaces in Chonburi Province, Thailand.

**Materials and Methods:**

A total of 244 macaques from Si Racha, Sattahip, and Mueang districts of Chonburi Province were sampled. Paired blood and oral mucosal swabs were collected from each animal and examined for SFV proviral DNA using nested polymerase chain reaction. Apparent prevalence was estimated, and potential risk factors were assessed using univariate and multivariable logistic regression. Agreement between sample types was evaluated using McNemar’s test and the Kappa coefficient (κ). Partial *pol* gene sequences from three SFV-positive samples were subjected to phylogenetic analysis against reference SFV sequences from different geographic regions.

**Results:**

Overall, 137/244 macaques were SFV-positive, corresponding to a prevalence of 56.15%. SFV prevalence was 32.79% in oral swabs and 36.89% in blood samples, with no significant difference between sample types. In univariate analysis, adult macaques had higher odds of SFV infection than juveniles (OR = 2.69; 95% confidence interval [CI] = 1.54–4.70; p < 0.001), while macaques weighing >5.0 kg had higher odds than those weighing <5.0 kg (OR = 2.84; 95% CI = 1.54–5.24; p < 0.001). In multivariable analysis, only body weight remained significantly associated with SFV infection (adjusted OR = 1.40; 95% CI = 1.15–1.71; p < 0.001), whereas age, sex, and geographic location were not significant predictors. Phylogenetic analysis showed that all three Thai SFV isolates clustered within a broader lineage containing SFV sequences from humans and non-human primates in Asia.

**Conclusion:**

SFV was highly prevalent among free-living long-tailed macaques in Chonburi Province, with body weight emerging as the principal independent factor associated with infection. The molecular findings further demonstrate the genetic relationship of Thai SFV isolates with viruses circulating among human and non-human primate hosts in Asia. These findings provide epidemiological and molecular baseline data for SFV surveillance and support continued One Health monitoring at human–macaque interfaces in Thailand.

## INTRODUCTION

The long-tailed macaque (*Macaca fascicularis*) is an Old World primate of the family Cercopithecidae that is widely distributed across Southeast Asia. As a highly adaptable, social species, it occupies diverse environments ranging from natural forests to urbanized areas and typically lives in structured social groups with well-defined dominance hierarchies. In Thailand, habitat loss, urban expansion, and intentional food provisioning have increased spatial overlap between macaque populations and human communities. These increasingly frequent interactions can result in human–macaque conflicts, including bites and scratches, which provide direct routes for zoonotic pathogen transmission [1, 2].

Simian foamy virus (SFV) is a complex retrovirus belonging to the subfamily Spumaretrovirinae and naturally infects all non-human primate (NHP) species investigated to date. Infection in NHPs is lifelong and generally considered non-pathogenic, with active viral replication occurring predominantly in superficial epithelial cells of the oral mucosa [3]. Although proviral DNA can be detected in several tissues and cell types, including peripheral blood mononuclear cells (PBMCs) [4], transmission occurs primarily through exposure to infected saliva, particularly through biting and other forms of close contact. Consequently, settings with frequent, direct human–macaque interactions may create opportunities for cross-species SFV exposure.

Zoonotic transmission of SFV has been documented among individuals with occupational and non-occupational exposure to NHPs, including bushmeat hunters, laboratory personnel, zoo workers, travelers, and communities living in close proximity to macaque populations [4–6]. Following cross-species transmission, SFV can integrate into the human host genome and establish persistent infection. Although sustained human-to-human transmission and overt SFV-associated clinical disease have not been confirmed, the ability of primate retroviruses to cross species barriers warrants continued surveillance. The emergence of human immuno-deficiency virus type 1 (HIV-1) from simian retroviral ancestors illustrates the broader importance of monitoring retroviral spillover at interfaces where humans and NHPs interact [7]. Emerging evidence further suggests that SFV infection may be associated with subtle physiological alterations and could influence disease progression in individuals co-infected with HIV-1 [8, 9].

Recent findings further emphasize SFV's One Health relevance. Neutralizing antibodies in chronically infected individuals may not completely prevent cell-to-cell viral transmission, potentially allowing viral persistence despite the host immune response [10]. Large-scale surveillance in Thailand has demonstrated a high prevalence of SFV among macaques, exceeding the prevalence of several other pathogens investigated in these populations [11]. Evidence from exposed human populations also indicates that NHP-derived SFV can persist following zoonotic transmission in communities experiencing prolonged contact with primates [12]. Furthermore, molecular analyses have demonstrated close phylogenetic relationships between zoonotic SFV strains and viruses detected in their source NHP hosts, suggesting limited viral adaptation following cross-species transmission [13]. Together, these findings reinforce the value of combining epidemiological surveillance with molecular characterization at human–NHP interfaces.

Despite growing recognition of SFV as an important virus at the human–animal interface, significant epidemiological and molecular knowledge gaps remain in Thailand. Existing surveillance indicates substantial SFV circulation among Thai macaques [11]; however, such broader prevalence estimates do not adequately characterize local infection patterns in individual macaque populations inhabiting areas with intensive human contact. In particular, site-specific epidemiological information from eastern Thailand, including Chonburi Province, remains limited despite the presence of free-living long-tailed macaque populations in urban and peri-urban environments where human–macaque interactions and conflicts occur. Consequently, the extent of SFV circulation, as well as the host and geographic factors associated with infection in these populations, remain insufficiently characterized.

A further limitation is the scarcity of molecular information on SFV circulating among free-living long-tailed macaques in Thailand. Epidemiological detection alone cannot establish the genetic relationships among locally circulating viruses or place them within the broader diversity of SFV strains identified in Asian human and NHP populations. In addition, SFV proviral DNA can be detected in both blood-derived cells and oral tissues, yet the relative detection rates and agreement between paired blood and oral swab specimens have not been sufficiently characterized in the target population. Addressing these gaps through paired-specimen detection, risk-factor analysis, and viral sequence characterization is important for improving understanding of SFV epidemiology at human–macaque interfaces and for establishing baseline information for future One Health surveillance.

Therefore, this study aimed to determine the prevalence of SFV infection and identify factors associated with SFV positivity among free-living *M. fascicularis* inhabiting human–macaque conflict areas in Chonburi Province, Thailand. The study further aimed to compare SFV detection rates and agreement between paired blood and oral swab specimens using nested polymerase chain reaction (PCR) and to molecularly characterize selected SFV isolates through partial *pol* gene sequencing and phylogenetic analysis. By integrating host-level epidemiological data, paired-specimen molecular detection, and phylogenetic characterization, the study sought to provide a more comprehensive understanding of SFV circulation in free-living long-tailed macaques and to generate baseline evidence relevant to One Health surveillance at human–macaque interfaces.

## MATERIALS AND METHODS

### Ethical approval

The study protocol was reviewed and approved by the Animal Ethics Committee of Rajamangala University of Technology Tawan-ok, Chonburi, Thailand (Permit No. RMUTTO ACUC 2 2023 010). All procedures involving capture, handling, anesthesia, biological sample collection, post-procedural monitoring, and release of free-living long-tailed macaques were conducted in accordance with institutional animal welfare requirements. Field procedures were performed by trained wildlife practitioners following the guidelines of the Department of National Parks, Wildlife and Plant Conservation [14]. During anesthesia and recovery, animals were monitored for heart rate, respiratory rate, body temperature, mucous membrane color, and recovery status. Following sample collection, macaques were monitored until full recovery and released at their original capture locations. The interval between capture and release did not exceed 24 h to minimize disruption of established troop social hierarchies.

## STUDY PERIOD AND LOCATION

The study was conducted from April to September 2023 in Chonburi Province, eastern Thailand. Sampling was undertaken in three districts: Si Racha (13.1425° N, 101.0485° E), Sattahip (12.6945° N, 100.9129° E), and Mueang (13.3625° N, 100.9807° E) (Figure 1). Specific sampling sites were Wat Phromawat, Bang Phra subdistrict, Si Racha; Naval Base Camp, Sattahip subdistrict, Sattahip; and Wat Khao Choeng Thian, Huay Kapi subdistrict, Mueang. The study was conducted as part of a macaque population-control program implemented by local administrative authorities.

**Figure 1:**
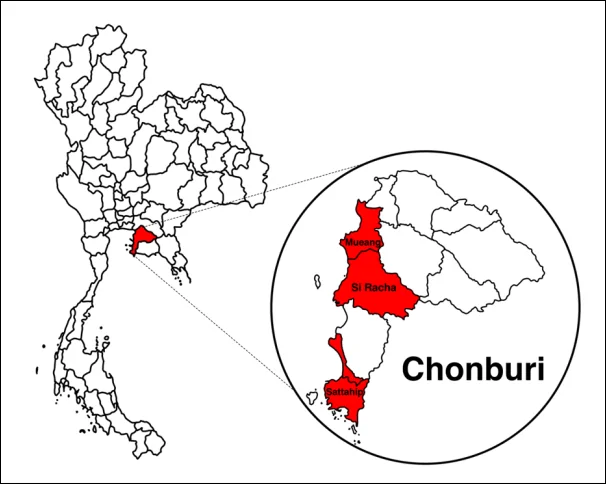
Map showing the study sites in Si Racha, Sattahip, and Mueang districts, Chonburi Province, Thailand [Source: The map was generated using Procreate version 5.3.4].

### Study design and sample size calculation

This cross-sectional study integrated field epidemiology, nested PCR, and molecular phylogenetic analysis to determine SFV prevalence, investigate associated risk factors, compare SFV detection between paired blood and oral swab specimens, and characterize selected SFV sequences in free-living *M. fascicularis*.

The required sample size was estimated using the single-population proportion formula, assuming an expected SFV prevalence of 12% based on the previous study by Nandi *et al.* [15], a 5% margin of error, and a 95% confidence interval (CI). The calculation was performed using EpiTools Epidemiological Calculators [16]. The intended number of samples from each study area was allocated proportionally according to local macaque density based on surveillance information and pre-visit assessments conducted by local administrative organization officers and regional veterinarians. However, the final number sampled at each site depended on the availability and physical condition of macaques during trapping. In total, 244 macaques were sampled, comprising 127 from Si Racha, 97 from Sattahip, and 20 from Mueang. Paired blood and oral swab specimens were obtained from each macaque.

### Animal capture, examination, and sample collection

Free-living macaques were captured using baited cages containing seasonal fruits, grains, and corn. Captured animals underwent physical examination before sedation. Macaques considered suitable for sampling were anesthetized by intramuscular administration of xylazine hydrochloride (0.5–2 mg/kg) and tiletamine–zolazepam (Zoletil™; 4.6 mg/kg; Virbac, Hamilton, New Zealand). During anesthesia, heart rate, respiratory rate, body temperature, mucous membrane color, and recovery status were monitored.

A minimum of 5.0 mL of blood was collected from the inguinal vein of each of the 244 anesthetized macaques and transferred into ethylenediaminetetraacetic acid (EDTA) tubes. An oral swab was also collected from each animal using a sterile cotton swab inserted into the mouth and rotated against the oral mucosa. Each swab was subsequently placed in an Eppendorf tube containing phosphate-buffered saline (PBS; pH 7.4).

The veterinary stations serving the sampling locations were approximately 7.2 km, 82.5 km, and 18 km from the university laboratory in Bang Phra for Si Racha, Sattahip, and Mueang, respectively. Transportation time was <2 h. During transport, specimens were maintained in insulated ice boxes at approximately 4°C and transferred to a −80°C freezer within 4 h of collection for further processing.

Body weight, body length, sex, and age category were recorded for each macaque. Animals were classified as adults or juveniles using a combination of body size, dental eruption and wear patterns, genital development, and muscular development, according to previously described criteria [17, 18].

Following sample collection, macaques were housed temporarily in cages and observed during recovery from anesthesia. Animals were released at their original capture locations after complete recovery. The total interval between capture and release did not exceed 24 h.

### Nested PCR for SFV detection

Genomic DNA was extracted from blood and oral swab specimens using the TIANamp® Genomic DNA Kit (Tiangen Biotech, Beijing, China) according to the manufacturer's instructions. DNA quality was evaluated using a NanoDrop 2000 spectrophotometer (Thermo Fisher Scientific, USA).

SFV proviral DNA was detected using nested PCR targeting a conserved region of the *pol* gene, based on the method described by Heneine *et al.* [19]. The primary and secondary primer sequences, annealing temperatures, number of amplification cycles, and expected product sizes are presented in Table 1. An SFV-positive control was obtained from the Faculty of Veterinary Science, Mahidol University, Salaya, Nakhon Pathom, Thailand [1], and a no-template control was included as the negative control in each PCR run.

**Table 1:** Oligonucleotide primers used for nested PCR amplification of the pol gene of simian foamy virus.

**Primer**	**Sequence (5′–3′)**	**Annealing temperature (°C)**	**No. of cycles**	**Product size (bp)**	**References**
SFV_pol_F1	GTGGNAAGGTGGAAAGGAAAAATAGTGANA	47	40	227	[1, 19]
SFV_pol_R1	NTANAGANNNNCNAATTTCCTGTAAAAGAGA	47	40	227	[1, 19]
SFV_pol_F2	NGTNGGNNGNCCTNCNAAGTGGTATGA	47	40	153	[1, 19]
SFV_pol_R2	NAANTCAAGTGTATCNNNNTTTGCAAANGG	47	40	153	[1, 19]

The same annealing temperature and number of cycles were used for the primary and secondary PCR reactions.

Thermal cycling conditions were adapted from previously published protocols [1, 19]. Amplification consisted of initial denaturation at 95°C for 2 min, followed by 40 cycles of denaturation at 94°C for 15 s, annealing at 47°C for 15 s, and extension at 72°C for 30 s, followed by a final extension at 72°C for 5 min. PCR products were separated by electrophoresis on 1.5% agarose gels and visualized under ultraviolet illumination using a gel imaging system (Daihan Scientific, Korea). SFV-positive samples selected for sequencing were confirmed by the presence of the expected 153-bp secondary PCR amplicon (Figure 2).

### Statistical analysis

Apparent SFV prevalence and corresponding 95% CIs were calculated for the overall study population and individual categories using Wilson binomial CIs. Associations between categorical variables and SFV positivity were initially evaluated using the χ² test, with statistical significance set at p < 0.05.

Univariate and multivariable logistic regression analyses were conducted to identify potential factors associated with SFV infection. Variables evaluated included sampling location (Si Racha, Sattahip, and Mueang), age category (adult or juvenile), sex (male or female), and body weight. For univariate analysis, body weight was categorized as >5.0 kg or 0–5.0 kg, and it was analyzed as a continuous variable in the multivariable logistic regression model. Associations are expressed as ORs with corresponding 95% CIs.

McNemar's test, with statistical significance set at p < 0.05, was used to compare SFV detection between paired blood and oral swab specimens. Agreement between the two specimen types was evaluated using Cohen's Kappa coefficient (κ). Agreement was classified as poor when κ < 0.40, good when κ = 0.40–0.75, and excellent when κ > 0.75 [20].

All statistical analyses except multivariable logistic regression were performed using EpiTools Epidemio-logical Calculators [16]. Multivariable logistic regression was performed using jamovi version 2.6.2.

### Molecular confirmation and phylogenetic analysis

Final PCR products from three SFV-positive blood samples (B001, B007, and B008) were selected for Sanger sequencing using previously published primers targeting the SFV *pol* gene [19]. These samples were selected because SFV was detected in both blood and oral swab specimens from the corresponding animals and sufficient DNA concentration and volume were available for downstream molecular analysis.

The resulting nucleotide sequences were compared with sequences available in GenBank using the Basic Local Alignment Search Tool (BLAST) of the National Center for Biotechnology Information (NCBI). Sequences generated in this study were deposited in GenBank under accession numbers PZ740612–PZ740617, with paired forward and reverse sequences submitted for each sample. Seventeen SFV *pol* gene sequences were purposively selected from the NCBI database to represent diverse geographic origins, NHP hosts, and human-derived SFV strains associated with primate exposure. Accession numbers and additional sequence information are provided in the supplementary data.

Phylogenetic analysis was performed using Geneious Prime version 2026.0.2. The three study sequences and 17 reference sequences were aligned using a minimum similarity threshold of 70%. A neighbor-joining phylogenetic tree was constructed using the Tamura–Nei genetic distance model, which accounts for differences in nucleotide substitution rates and base frequencies. Tree reliability was evaluated using 1,000 bootstrap replicates. No outgroup was designated. The resulting phylogenetic tree was edited and visualized using Canva Pro.

## RESULTS

### Prevalence of SFV

The overall prevalence of SFV infection among free-living macaques in Chonburi Province, Thailand, was 56.15% (137/244; Table 2). Paired blood and oral swab specimens were tested from each macaque, and an animal was classified as SFV-positive (Figure 2) when viral DNA was detected in at least one specimen type. Among the 244 macaques, 90 blood samples (36.89%) and 80 oral swab samples (32.79%) tested positive for SFV by nested PCR (Table 3). Thirty-three macaques tested positive in both specimen types, 57 were positive only in blood, 47 were positive only in oral swabs, and 107 were negative in both specimen types. Individual-level results are provided in the supplementary data.

**Table 2:** Overall prevalence, univariate analysis, and multivariable logistic regression of risk factors associated with simian foamy virus infection in free-living macaques in Chonburi Province, Thailand, 2023.

**Risk-factor**	**Category**	**No. of samples**	**Prevalence, % (95% CI)**	**Univariate OR (95% CI)**	**p-value**	**Adjusted OR (95% CI)**	**p-value**
Habitat	Si Rachaᵃ	127	55.91 (47.2–64.2)	0.98 (0.59–1.62)	0.940	—	—
	Sattahip	97	56.70 (46.8–66.1)	1.04 (0.62–1.74)	0.890	1.03 (0.16–6.79)	0.974
	Mueang	20	55.00 (34.2–74.2)	0.95 (0.38–2.38)	0.914	1.01 (0.57–1.79)	0.970
Age	Adult	168	63.69 (56.2–70.6)	2.69 (1.54–4.70)	<0.001*	1.15 (0.53–2.46)	0.730
	Juvenile	76	39.47 (29.3–50.7)	—	—	—	—
Sex	Male	154	56.49 (48.6–64.1)	1.04 (0.62–1.75)	0.887	0.56 (0.29–1.10)	0.091
	Female	90	55.56 (45.3–65.4)	—	—	—	—
Body weightᵇ	>5.0 kg	71	73.24 (62.0–82.2)	2.84 (1.54–5.24)	<0.001*	1.40 (1.15–1.71)	<0.001*
	0–5.0 kg	157	49.04 (41.3–56.8)	—	—	—	—
Overall		244	56.15 (49.9–62.2)	—	—	—	—

CI = Confidence interval; OR = Odds ratio. ᵃSi Racha = Reference category for habitat in the multivariable logistic regression analysis. ᵇBody-weight data = Unavailable for 16 macaques. *p < 0.05.

Comparison of SFV detection between paired blood and oral swab specimens is presented in Table 3. Overall, McNemar’s test showed no significant difference in detection between blood and oral swab samples (p = 0.378). However, specimen-specific detection differed significantly in Sattahip (p = 0.027) and among juvenile macaques (p = 0.021). Overall agreement between blood and oral swab results was poor (κ = 0.06), with an overall percentage agreement of 57.38%.

**Table 3:** Comparison of simian foamy virus prevalence detected in blood and oral swab samples from free-living macaques in Chonburi Province, Thailand, 2023 (n = 244).

**Category**	**Blood positive, n (%)**	**Oral swab positive, n (%)**	**McNemar’s p-value**	**κ**	**Agreement (%)**
Habitat					
Si Racha	45 (35.43)	47 (37.01)	0.888	0.15	60.63
Sattahip	40 (41.24)	24 (24.74)	0.027*	0.04	52.58
Mueang	5 (25.00)	9 (45.00)	0.289	0.16	60.00
Age					
Adult	67 (39.88)	70 (41.67)	0.820	0.05	54.14
Juvenile	23 (30.26)	10 (13.16)	0.021*	0.00	64.47
Sex					
Male	62 (40.26)	46 (29.87)	0.065	0.07	57.14
Female	28 (31.11)	34 (37.78)	0.413	0.07	57.78
Body weightᵃ					
>5.0 kg	38 (53.52)	32 (45.07)	0.391	0.05	52.11
0–5.0 kg	48 (30.57)	42 (26.75)	0.525	0.03	60.51
Overall	90 (36.89)	80 (32.79)	0.378	0.06	57.38

Percentages were calculated using the total number of macaques in each category as the denominator. ᵃBody-weight data = Unavailable for 16 macaques. κ = Kappa coefficient. *p < 0.05.

**Figure 2:**
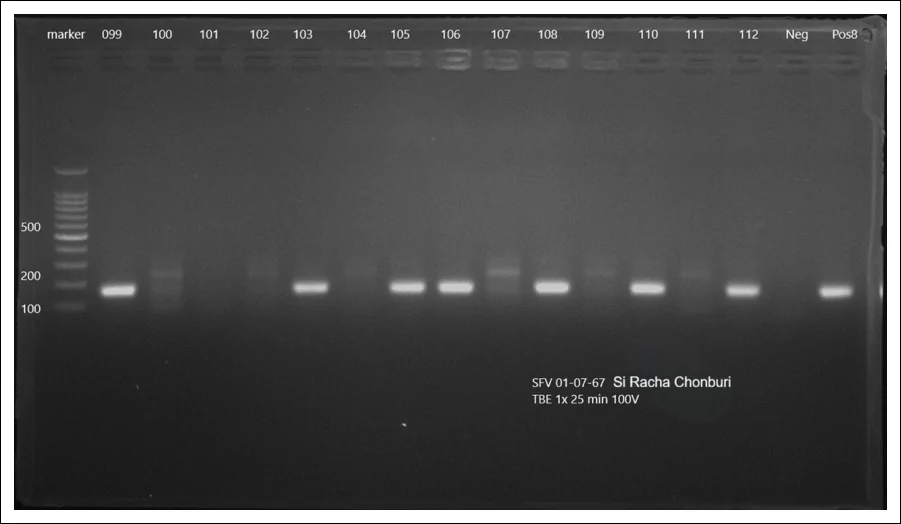
Representative nested PCR amplification of the simian foamy virus *pol* gene in blood samples from free-living *Macaca fascicularis* in Chonburi Province, Thailand. The expected 153-bp amplicon was detected in representative macaque samples. Lanes 099–112 = Representative individual samples; M = DNA marker; Neg = No-template negative control; Pos = Positive control. PCR products were resolved on a 1.5% agarose gel and visualized under ultraviolet illumination.

### Risk factors associated with SFV infection

Univariate analysis showed that adult macaques had a significantly higher SFV prevalence than juveniles (63.69% vs. 39.47%; OR = 2.69; 95% CI = 1.54–4.70; p < 0.001). Body weight was also significantly associated with SFV infection in the univariate analysis. Macaques weighing >5.0 kg had a prevalence of 73.24%, compared with 49.04% among those weighing 0–5.0 kg (OR = 2.84; 95% CI = 1.54–5.24; p < 0.001).

In the multivariable logistic regression analysis, body weight remained significantly associated with SFV infection (adjusted OR = 1.40; 95% CI = 1.15–1.71; p < 0.001), whereas age was no longer significantly associated with infection after adjustment for the other variables (adjusted OR = 1.15; 95% CI = 0.53–2.46; p = 0.730). Body weight data were unavailable for 16 macaques; therefore, these animals were excluded from analyses involving body weight (Table 2).

SFV prevalence did not differ significantly according to geographic location. Prevalence was 55.91% in Si Racha, 56.70% in Sattahip, and 55.00% in Mueang, with no significant associations detected in either univariate or multivariable analyses. Sex was also not significantly associated with SFV infection; prevalence was 56.49% among males and 55.56% among females (Table 2).

### Phylogenetic analysis of SFV

Phylogenetic analysis based on partial *pol* gene sequences showed that the three Thai SFV isolates obtained from macaques in Chonburi Province (B001, B007, and B008) clustered together within a single lineage and were most closely related to a macaque-derived SFV sequence from India (GQ472896/2009) (Figure 3).

The Thai isolates grouped within a broader lineage containing previously reported SFV sequences derived from macaques and humans in Asia and Africa. In contrast, several South American and African primate-derived sequences formed distinct lineages. These results demonstrate the phylogenetic relationships of the three Chonburi SFV isolates with previously reported SFV sequences from geographically and host-diverse sources.

**Figure 3:**
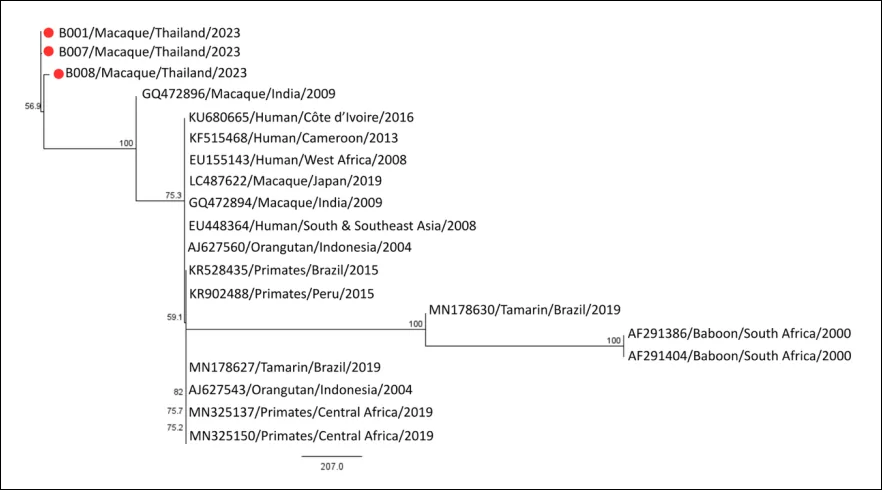
Neighbor-joining phylogenetic tree based on partial *pol* gene sequences of simian foamy virus. The tree was constructed using the Tamura–Nei genetic distance model with 1,000 bootstrap replicates. Values at the nodes represent bootstrap support percentages. Thai SFV isolates from Chonburi Province (B001, B007, and B008) are indicated by red circles. Reference sequences retrieved from GenBank are labeled with their accession numbers. Phylogenetic analysis was performed using Geneious Prime version 2026.0.2.

## DISCUSSION

This study integrated epidemiological and molecular approaches to investigate SFV infection in free-living long-tailed macaques at human–macaque interfaces in eastern Thailand. In Thailand, many habitats occupied by *M. fascicularis* overlap with human communities, creating repeated opportunities for contact and potential bidirectional pathogen exposure [1, 2, 11]. The overall SFV prevalence of 56.15% observed in the present study indicates substantial viral circulation among macaque populations in Chonburi Province, which includes both natural and semi-urban environments. This prevalence is comparable with previous reports from Thailand, in which approximately 52%–56% of free-living macaques were SFV-positive [1, 11].

### SFV prevalence in free-living macaques

High SFV prevalence has also been reported in free-living macaque populations elsewhere in South and Southeast Asia. In Cambodia, PCR-based and serological investigations detected SFV infection rates of 15% and 45%, respectively [21], whereas prevalence estimates of 90%–100% have been reported in Singapore [22]. Similarly, SFV infection was detected in 97.4% of temple macaques in Nepal [23] and in 98% of free-living rhesus macaques in India [24]. Outside South and Southeast Asia, SFV surveillance has more frequently involved captive NHP populations in research facilities, zoological collections, or reserves. Reported PCR-based prevalence estimates include 26.9% in China [25], 34.8% in Brazil [26], and 44.5% in Gabon [27], whereas seroprevalence estimates of 62%–100% have been reported in Germany [28]. Collectively, these studies demonstrate the widespread occurrence of SFV in primate populations, although prevalence estimates vary considerably according to host species, ecological setting, study design, and diagnostic approach.

Serological and molecular methods are among the principal approaches used for SFV detection [29], but the method selected can influence prevalence estimates. Several studies have reported higher prevalence when SFV exposure was assessed serologically than when proviral DNA was detected by PCR [11, 21, 22]. Serological assays primarily reflect cumulative exposure and persistent host antibody responses, whereas PCR detection depends on the presence and abundance of proviral DNA in the sampled biological material. Consequently, variation between studies may partly reflect differences in assay type, specimen characteristics, timing of collection, and viral distribution within host tissues [11].

### Comparison of blood and oral swab specimens

The biological specimen used for molecular testing may also influence SFV detection. In the present study, SFV prevalence was slightly higher in blood samples than in oral swabs, although the overall difference between specimen types was not statistically significant (p = 0.378). SFV persists as proviral DNA in circulating leukocytes, supporting molecular detection from blood even when active viral replication is limited [4]. In contrast, oral swabs sample the oral compartment, where active SFV replication and salivary shedding may vary over time [22, 30].

Agreement between paired blood and oral swab results was poor (κ = 0.06). Of the 244 macaques, 33 tested positive in both specimen types, whereas 57 were positive only in blood and 47 only in oral swabs. This substantial discordance indicates that a single specimen type may not identify all infected animals detected through paired sampling. Differences in viral burden, specimen quality, cellular content, and availability of the PCR target may have contributed to the observed discordance. Biological compartmentalization of SFV may also be relevant, although the present study was not designed to directly evaluate tissue-specific viral dynamics.

Despite the poor individual-level agreement, overall prevalence estimates obtained from blood and oral swabs did not differ significantly. Therefore, both specimen types may provide useful information for population-level surveillance, although they should not necessarily be regarded as interchangeable for individual diagnosis. Selection of specimen type in field studies should consider sampling feasibility, animal welfare, invasiveness, sample quality, and the specific epidemiological objective.

### Age and body weight as factors associated with SFV infection

Age was significantly associated with SFV infection in the univariate analysis, with adults showing a higher prevalence than juveniles. However, this association was no longer significant after adjustment for other variables in the multivariable model. The loss of significance may partly reflect the close biological relationship between age and body weight, as these variables are not independent in growing macaques. The use of categorical age classification may also reduce the ability to distinguish more gradual age-related changes in exposure.

The higher unadjusted prevalence among adults is nevertheless consistent with previous reports demonstrating increasing SFV infection with age [11, 21, 26]. Older macaques have had more time to encounter infected conspecifics, increasing cumulative opportunities for exposure [24, 31]. Adult animals may also participate more frequently in aggressive and dominance-related interactions, including biting, scratching, and fighting, which can facilitate transmission through infected saliva [32]. However, behavioral interactions were not directly quantified in this study, and therefore the contribution of aggressive behavior cannot be confirmed from the present data.

Body weight was significantly associated with SFV infection in both univariate and multivariable analyses. Macaques weighing >5.0 kg had a substantially higher prevalence than lighter animals, and body weight remained significantly associated with SFV positivity after adjustment for other variables. Because body weight is closely related to age and physical maturity in long-tailed macaques, this association may partly reflect cumulative exposure over time rather than an independent biological effect of body mass itself. Larger animals may also differ in dominance status and social interaction patterns, although these variables were not directly measured.

The 5.0-kg threshold used in the univariate analysis was supported by external morphological characteristics, including body size and dental development [17, 18]. Body weight may therefore serve as a practical field indicator associated with SFV infection risk. Nevertheless, body weight data were unavailable for 16 macaques, and the possibility of bias resulting from these missing observations should be considered when interpreting the strength of this association.

### Sex and geographic location

Sex was not significantly associated with SFV infection, which is consistent with previous studies [11, 25, 26]. Although male macaques may engage more frequently in aggressive interactions, the social organization of macaque troops creates extensive contact among animals of both sexes and multiple age classes. Because SFV transmission is closely associated with direct contact and exposure to infected bodily fluids [25, 32], shared social environments may reduce detectable differences in infection prevalence between males and females.

Geographic location was also not significantly associated with SFV infection. Prevalence was similar among macaques sampled in Si Racha, Sattahip, and Mueang despite ecological differences among the three districts. Macaques in Mueang and Si Racha predominantly occupy urban or semi-urban settings and frequently exploit human-associated food resources, whereas those in Sattahip are more commonly associated with natural habitats and only intermittently enter human settlements.

The absence of a significant geographic effect suggests that SFV is widely distributed among the sampled macaque populations in Chonburi Province. However, this finding should not be interpreted as evidence that habitat or human contact has no influence on transmission. The number of macaques sampled differed considerably among districts, particularly in Mueang, and detailed behavioral or ecological exposure variables were not incorporated into the analysis. Larger studies incorporating direct measures of human contact, macaque density, troop structure, resource provisioning, and aggressive interactions would be required to determine whether ecological conditions modify SFV transmission at a finer spatial scale.

### Molecular epidemiology and phylogenetic relationships

Molecular information on SFV circulating in free-living Thai macaques remains limited. In the present study, partial *pol* gene sequencing was performed on three confirmed SFV-positive samples to provide preliminary molecular characterization. The three Thai isolates clustered together and were most closely related to a macaque-derived SFV sequence from India. They also grouped within a broader lineage containing SFV sequences derived from macaques and humans from Asia and Africa.

These findings expand the available molecular evidence for SFV in free-living Thai macaques and provide preliminary information on the phylogenetic placement of viruses circulating in Chonburi Province. The separation of the Thai isolates from several South American and African primate-derived lineages is consistent with previous studies demonstrating geographic and host-associated structuring of SFV populations [33, 34]. Such structuring is compatible with the long-term co-evolution of foamy viruses with their primate hosts.

The clustering of Thai macaque-derived sequences within a broader lineage that also contains human-derived SFV sequences is compatible with the established zoonotic potential of SFV [13, 23, 33]. However, phylogenetic clustering alone cannot demonstrate direct transmission between the macaques sampled in the present study and humans. No human samples were collected, and no epidemiologically linked human–macaque transmission events were investigated. Therefore, the phylogenetic findings should be interpreted as evidence of evolutionary relatedness rather than direct proof of cross-species transmission in Chonburi.

### Utility and limitations of the *pol* gene marker

The *pol* gene was selected because of its relatively high conservation among SFV strains, which supports reliable molecular detection and broad phylogenetic comparison across different primate hosts [35]. Functional constraints on *pol* contribute to comparatively low sequence variability, making this genomic region useful for lineage-level classification. However, the same conservation can limit discrimination among closely related strains and reduce resolution for fine-scale evolutionary or transmission analyses [34].

Previous studies have therefore used additional genomic regions, including long terminal repeats and combinations of *gag*, *pol*, and *env*, to improve strain discrimination and strengthen phylogenetic inference [13, 36]. Future analyses incorporating longer genomic regions or whole-genome sequencing could provide greater resolution of SFV diversity in Thai macaques and allow more robust investigation of geographic and host-associated viral lineages.

### Strengths and limitations

A major strength of this study was the integration of field epidemiology, paired-specimen molecular detection, risk-factor analysis, and phylogenetic characterization within the same free-living macaque populations. The inclusion of 244 macaques across three districts also provided a substantial dataset for estimating SFV prevalence in Chonburi Province. Paired blood and oral swab sampling further enabled direct comparison of specimen-specific molecular detection within individual animals.

Several limitations should nevertheless be considered. First, the cross-sectional design precluded assessment of SFV incidence, acquisition, persistence, and temporal changes in viral shedding. Second, sampling was restricted to selected locations within Chonburi Province, and the markedly smaller sample from Mueang may have reduced statistical power for geographic comparisons. Consequently, the findings may not represent SFV epidemiology in all free-living macaque populations in Thailand.

Third, the absence of serological testing limited assessment of cumulative exposure and prevented direct comparison of molecular and antibody-based estimates within the same animals. Human participants were also not sampled; therefore, zoonotic transmission at the investigated human–macaque interfaces could not be directly assessed. Fourth, body weight data were unavailable for 16 macaques, which may have influenced analyses involving this variable.

Finally, molecular characterization was based on only three isolates and a short partial *pol* gene fragment. This limited sample is unlikely to capture the full genetic diversity of SFV circulating in the study populations and restricts fine-scale phylogenetic inference. The absence of broader genomic characterization similarly limits conclusions regarding strain diversity, transmission chains, or local viral evolution.

### Future research perspectives

Future studies should employ longitudinal surveillance to characterize SFV acquisition, persistence, and temporal patterns of oral shedding. Expanded geographic sampling across additional macaque populations in Thailand would help determine whether the patterns observed in Chonburi Province are representative of other regions. Combining molecular detection with serological testing would provide a more complete assessment of both current proviral detection and cumulative exposure.

More comprehensive molecular approaches, including sequencing of additional genomic regions or whole-genome sequencing, are also warranted to improve phylogenetic resolution and characterize local SFV diversity. Incorporating detailed ecological and behavioral variables, including troop structure, population density, aggressive interactions, provisioning, and frequency of direct human contact, could further clarify the determinants of SFV transmission. Finally, carefully designed One Health studies that include both macaque and appropriately exposed human populations would be required to directly investigate zoonotic transmission pathways at human–macaque interfaces.

## CONCLUSION

This study demonstrated a high prevalence of SFV infection among free-living long-tailed macaques in Chonburi Province, Thailand, with 137 of 244 animals testing positive, corresponding to an overall prevalence of 56.15%. SFV was detected in 36.89% of blood samples and 32.79% of oral swab samples, with no significant overall difference between specimen types, although agreement was poor (κ = 0.06). Adult macaques and those weighing >5.0 kg showed higher SFV prevalence in univariate analysis, but body weight remained the only significant factor in the multivariable model (adjusted OR = 1.40; 95% CI = 1.15–1.71; p < 0.001). Sex and geographic location were not significantly associated with infection. Partial *pol* gene analysis further showed that the three Thai SFV isolates clustered together and were most closely related to a macaque-derived SFV sequence from India, within a broader lineage containing SFV sequences from macaques and humans in Asia and Africa.

From a practical perspective, these findings support continued SFV surveillance in free-living macaque populations inhabiting areas with frequent human contact. The poor agreement between blood and oral swab results indicates that specimen choice may influence individual-level detection and should therefore be considered when designing field surveillance programs. Body weight may also serve as a useful field-associated indicator for identifying macaques with a higher probability of SFV positivity, although it should not be interpreted independently of age and other host characteristics. The molecular findings provide additional baseline information for monitoring SFV diversity and regional phylogenetic relationships in Thailand.

Overall, the integration of epidemiological, paired-specimen, and molecular data provides a more comprehensive understanding of SFV circulation in free-living macaques at human–macaque interfaces in eastern Thailand. These findings reinforce the importance of One Health-based surveillance strategies that combine wildlife monitoring with molecular characterization. Continued surveillance and broader molecular investigations will be important for clarifying SFV transmission dynamics and improving risk assessment at interfaces where humans and macaques coexist.

## DATA AVAILABILITY

All data generated or analyzed during this study are included in this published article, its supplementary information files, and the GenBank database under accession numbers PZ740612-PZ740617. The supplementary data can be obtained from the corresponding author upon a request.

## GENERATIVE AI DECLARATION

The authors declare that generative artificial intelligence (AI) tools were used solely to improve language, grammar, and readability during manuscript preparation. All scientific content, data analysis, interpretation of results, and conclusions were developed and verified by the authors. The authors take full responsibility for the accuracy, integrity, and originality of the work presented, and no AI tool was listed as an author.

## AUTHORS’ CONTRIBUTIONS

DT: Study design, sample collection, and writing – original draft, review, and editing. PP: Sample collection, coordination with local authorities, data analysis, and writing – original draft. PM: Sample collection, organization of field activities, and coordination with local authorities. SK: Laboratory analysis, molecular detection, and sequence analysis. PK: Laboratory analysis, molecular detection, and sequence analysis. WN: Sample collection, statistical analysis, and visualization. SP: Conceptualization, study design, data analysis, and writing – original draft, review, and editing. All authors have read and approved the final version of the manuscript.
